# Characterization of *biklf*/*klf17*-deficient zebrafish in posterior lateral line neuromast and hatching gland development

**DOI:** 10.1038/s41598-019-50149-5

**Published:** 2019-09-26

**Authors:** Hiroaki Suzuki, Tomoe Ishizaka, Kanoko Yanagi, Ryota Sone, Yuto Sunaga, Rie Ohga, Atsuo Kawahara

**Affiliations:** 1 Laboratory for Developmental Biology, Center for Medical Education and Sciences, Graduate School of Medical Science, University of Yamanashi, Shimokato 1110, Chuo, Yamanashi 409-3898 Japan; 2 Department of Pediatrics, Faculty of Medicine, University of Yamanashi, Shimokato 1110, Chuo, Yamanashi 409-3898 Japan

**Keywords:** Embryogenesis, Organogenesis

## Abstract

Krüpple-like factors (Klfs) are highly conserved zinc-finger transcription factors that regulate various developmental processes, such as haematopoiesis and cardiovascular development. In zebrafish, transient knockdown analysis of *biklf*/*klf17* using antisense morpholino suggests the involvement of *biklf*/*klf17* in primitive erythropoiesis and hatching gland development; however, the continuous physiological importance of *klf17* remains uncharacterized under the genetic ablation of the *klf17* gene among vertebrates. We established the *klf17*-disrupted zebrafish lines using the CRISPR/Cas9 technology and performed phenotypic analysis throughout early embryogenesis. We found that the *klf17*-deficient embryos exhibited abnormal lateral line neuromast deposition, whereas the production of primitive erythrocytes and haemoglobin production were observed in the *klf17*-deficient embryos. The expression of lateral line neuromast genes, *klf17* and *s100t*, in the *klf17*-deficient embryos was detected in posterior lateral line neuromasts abnormally positioned at short intervals. Furthermore, the *klf17*-deficient embryos failed to hatch and died without hatching around 15 days post-fertilization (dpf), whereas the dechorionated *klf17*-deficient embryos and wild-type embryos were alive at 15 dpf. The *klf17*-deficient embryos abolished hatching gland cells and Ctsl1b protein expression, and eliminated the expression of polster and hatching gland marker genes, *he1*.*1*, *ctsl1b* and *cd63*. Thus, the *klf17* gene plays important roles in posterior lateral line neuromast and hatching gland development.

## Introduction

Krüpple-like transcription factors (Klfs), which are characterized by the Cys2-His2 zinc-finger motif at the C-terminus, are involved in various developmental processes, such as haematopoiesis and cardiovascular development^1,2^. Phylogenetic analysis suggests that the Klf17 family belongs to a distinct branch closely related to Klf2 and Klf4 families, and mammalian Klf17 proteins remarkably diverged from those of other species^3^, showing rapid evolution in mammals. In mice, the *Klf17* gene was identified as a germ cell-specific gene^4^. Recent accumulating evidence suggests that the *Klf17* gene in mammals plays important roles in tumourigenesis^5^. The *Klf17* gene is down-regulated in various human cancers^6^, presumably leading to the epithelial-mesenchymal transition (EMT) and metastases. Importantly, the physiological function of *Klf17* during mammalian embryogenesis is not fully understood.

*klf17*/*biklf* (*blood island*-*enriched Krüpple*-*like factor*) was originally identified as one of the zygotic-activated genes during zebrafish embryogenesis^7^. Zebrafish *klf17* is expressed in the involuting axial mesoderm and polster during gastrulation and is subsequently expressed in the blood island/intermediate cell mass (ICM), hatching gland and lateral lines during organogenesis. *Xenopus klf17*/*neptune* is expressed in the hatching gland, cement gland and ventral blood island during embryogenesis^8,9^, whereas the chick *Klf17* gene is expressed in the blood island^10^. Knockdown of zebrafish *klf17* gene results in the impairment of primitive erythropoiesis and of hatching gland development^11–13^. The *Xenopus klf17*/*neptune* morphants exhibit loss of the hatching gland and otic vesicle and malformation of neural crest-derived cranial cartilage^14^.

Recent studies demonstrate that genetically gene-disrupted mutants and morphants induced by transient morpholino injection often exhibit distinct morphological phenotypes^15^. In fact, morpholino injection in zebrafish embryos often causes ectopic p53 induction^16^. Furthermore, undesirable off-target effects mediated by morpholino injection have been reported^17^. Transient knockdown analysis using morpholino injection is unsuitable for continuous *in vivo* analysis; therefore, we have generated the *klf17*-disrupted zebrafish mutants using the CRISPR/Cas9 technology and examined the continuous physiological function of *klf17* throughout early embryogenesis.

## Results

### Primitive haematopoiesis in the *klf17*-deficient embryos

To examine the physiological function of *klf17* during zebrafish early embryogenesis, we established the *klf17*-disrupted zebrafish lines using the genome editing technology, CRISPR/Cas9. Three *klf17* alleles *uy21*, *uy22* and *uy23* with totally 20 base pairs (bp), 20 bp deletions and a 28 bp insertion, respectively, were isolated (Supplemental Figs S1 and S2). Because the predicted Klf17 mutant proteins derived from mutant alleles lacked most of the coding domains including zinc fingers (Supplemental Fig. S3), they would be functionally disrupted.

**Figure 1:**
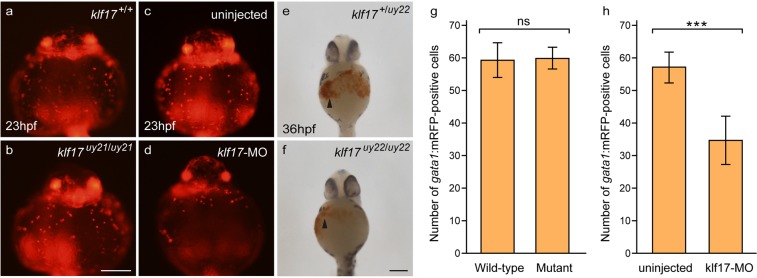
Primitive erythrocytes were observed in the *klf17*-deficient embryos. (**a**–**d**) Production of primitive erythrocytes in the *klf17*-deficient embryo and in the *klf17*-morphant embryo at 23 hpf. The number of erythrocytes visualized by the *gata1*:mRFP transgene was comparable in wild-type embryo (*klf17*^+/+^) (**a**) and the *klf17*-deficient embryo (*klf17*^*uy21*/*uy21*^) (**b**) (ventral view, anterior up), whereas the number of erythrocytes was less in the *klf17*-morphant embryo (**d**). Scale bar, 200 μm. (**e**,**f**) Haemoglobin production in the *klf17*-deficient embryos at 36 hpf. Haemoglobin production visualized by *o*-dianisidine staining (arrowheads) was detected in the wild-type embryo containing an intact allele (*klf17*^+/*uy22*^) embryo and in the *klf17*-deficient embryo (*klf17*^*uy22*/*uy22*^) (ventral view, anterior up). After taking pictures, genotyping of individual embryos was performed by genomic PCR. Scale bar, 200 μm. (**g**) The number of *gata1*:mRFP-positive cells on the yolk in wild-type (n = 19) and in the *klf17*-deficient embryos (mutant) (n = 8) were counted. Error bars indicate standard deviation. ns, not significant. (**h**) The number of *gata1*:mRFP-positive cells on the yolk in uninjected (n = 14) and in the *klf17*-molpholino (10 ng)-injected embryos (n = 19) were counted. Asterisk indicates statistical significance. ***P < 0.001. Error bars indicate standard deviation.

Knockdown analysis of the *klf17* gene using antisense morpholino suggests that the zebrafish *klf17* gene is involved in primitive erythropoiesis development^11–13^. Therefore, we first examined the production of primitive erythrocytes monitored by *gata1*:mRFP transgene and haemoglobin production by *o*-dianisidine staining in the *klf17*-deficient embryos. The number of erythrocytes on the yolk seemed to be comparable in wild-type (n = 19) and *klf17*-deficient embryos (n = 8) at 23 hours post-fertilization (hpf), whereas primitive erythrocytes were decreased in the *klf17*-morphant (n = 19) (Fig. 1). Production of haemoglobin and circulating blood cells were detected in the *klf17*-deficient embryos (n = 10) and in wild-type embryos containing intact alleles (n = 8) at 36 hpf (Figs 1, S4 and Supplemental Movies 1–3). Furthermore, the expression of erythrocyte genes, *gata1*, *β*_*e3*_*globin* (an embryonic globin) at 18-somite and 25 hpf was comparable in wild-type and *klf17*-deficient embryos (Supplemental Fig. S5). The expression of myeloid cell marker *lysozyme C* (*lyz*) was comparably expressed in wild-type and the *klf17*-deficient embryos at 25 hpf. Thus, we did not observe the severe impairment of primitive erythropoiesis reported in the analysis of the *klf17*-morphant^11,13^.

### Lateral line neuromast development in the *klf17*-deficient embryos

**Figure 2:**
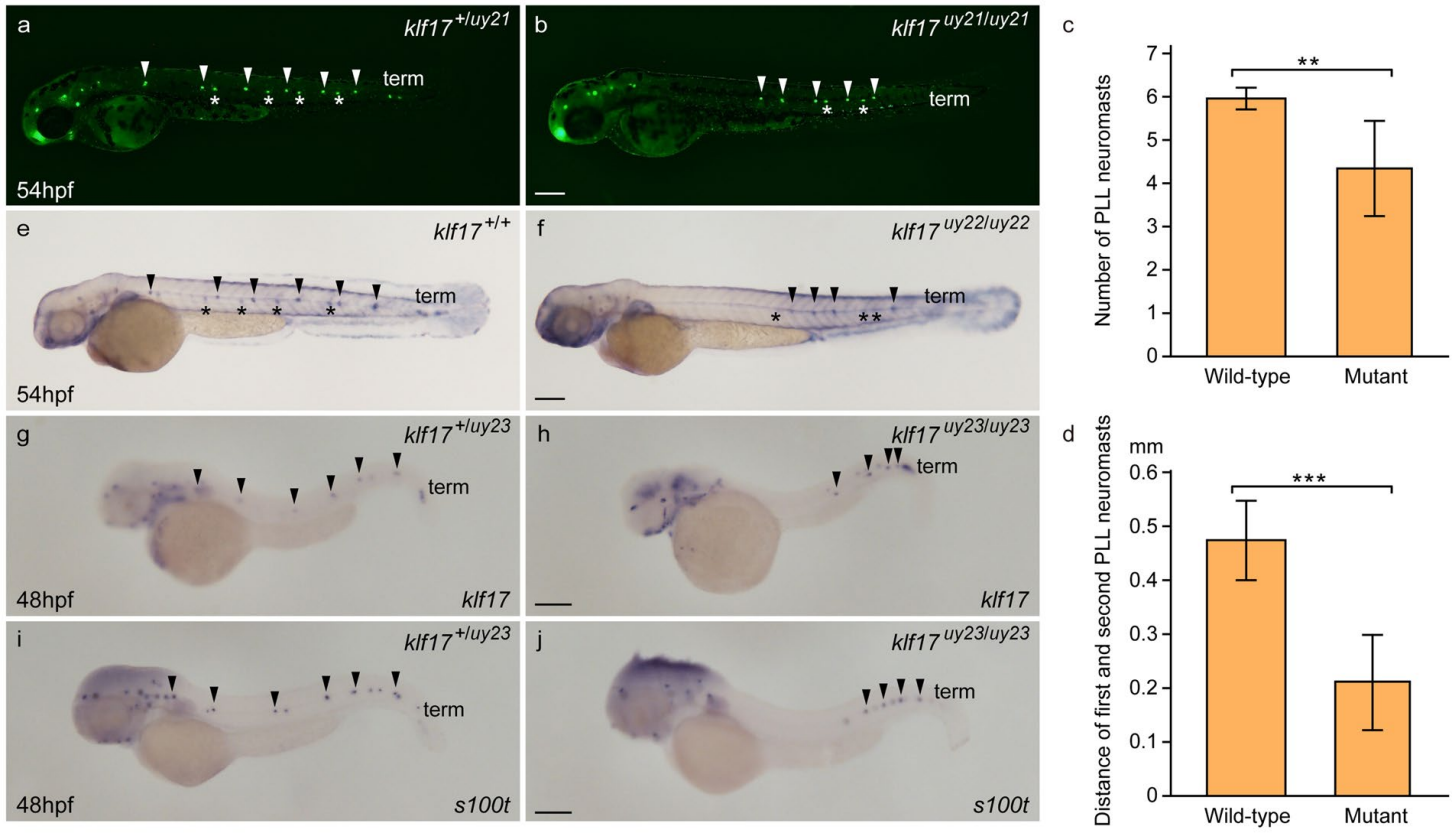
Abnormal PLL neuromast deposition in the *klf17*-deficient embryos. (**a**–**d**) Lateral line neuromasts stained with 4-Di-2-ASP (Di-ASP). PLL neuromasts were bilaterally stained with Di-ASP. White arrowheads indicate the position of PLL neuromasts on the left side. White asterisks indicate the position of PLL neuromasts on the right side. Term indicates the position of terminal neuromasts. The number of PLL neuromasts (**c**) and the distance between first and second neuromasts (**d**) were measured in wild-type (n = 16) and the *klf17*-deficient embryos (n = 9). Asterisk indicates statistical significance between wild-type and the *klf17*-deficient embryos. **P < 0.01. ***P < 0.001. Error bars indicate standard deviation. (**e**,**f**) Alkaline phosphatase accumulation in the lateral line neuromasts. Arrowheads indicate the position of PLL neuromasts on the left side. Asterisks indicate the position of PLL neuromasts on the right side. (**g**–**j**) The expression of lateral line genes, *klf17* (**g**,**h**) and *s100t* (**i**,**j**), was examined by whole-mount *in situ* hybridization (WISH) at 48 hpf. (**g**,**i**) Wild-type embryos (*klf17*^+/*uy23*^). (**h**,**j**) *klf17*-deficient embryos (*klf17*^*uy23*/*uy23*^). Arrowheads indicate the position of PLL neuromasts on the left side. All pictures showed lateral view, anterior left. After taking pictures, genotyping of individual embryos was performed by genomic PCR. Scale bar, 200 μm.

Because *klf17* gene is bilaterally expressed in the lateral line neuromasts^18^, we examined the differentiation of lateral line neuromasts in the *klf17*-deficient embryos at 54 hpf using the fluorescent reagent 4-Di-2-ASP (Di-ASP) that can easily label differentiated neuromasts^19^. The posterior lateral line (PLL) primordium migrated caudally and periodically deposited neuromasts at regular five or six intervals and two or three terminal neuromasts (term) along the horizontal myoseptum of wild-type embryos. Deposition of the first PLL neuromast in the *klf17*-deficient embryos was posteriorly delayed (Fig. 2). The number of PLL neuromasts in the *klf17*-deficient embryos (n = 9) was less compared to that of wild-type (n = 16). The distance between first and second PLL neuromasts was short in the *klf17*-deficient embryos. Using visualization of alkaline phosphatase accumulation, the delay of the PLL neuromasts deposition was observed in the *klf17*-deficient embryos (n = 5) compared to wild-type embryos (n = 15) (Fig. 2e,f). Injection of *klf17* mRNA (20 pg) in wild-type and the *klf17*-deficient embryos caused to axis defects at 36 hpf (Supplemental Fig. S6). The expression of lateral line neuromast genes, *klf17* and *s100t*, in the PLL neuromasts was located posteriorly in the *klf17*-deficient embryos (Fig. 2g–j). Thus, the *klf17* gene is required for proper PLL neuromasts deposition.

### Hatching gland development in the *klf17*-deficient embryos

**Figure 3:**
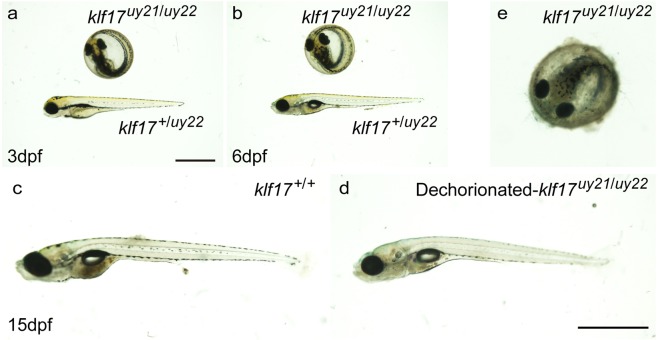
*klf17*-deficient embryos failed to hatch during zebrafish embryogenesis. (**a**,**b**) Hatching-deficient phenotype in the *klf17*-deficient embryos at 3 dpf (**a**) and 6 dpf (**b**). The *klf17*-deficient embryo (*klf17*^*uy21*/*uy22*^) failed to hatch at 3 and 6 dpf, whereas wild-type (*klf17*^+/*uy22*^) embryo hatched until 3 dpf. Scale bar, 1 mm. (**c**–**e**) Dechorionated-*klf17*-deficient embryos were alive at 15 dpf. Wild-type embryo (*klf17*^+/+^) (**c**) and the dechorionated- *klf17*-deficient embryo (*klf17*^*uy21*/*uy22*^) (**d**) were both alive at 15 dpf, whereas the *klf17*-deficient embryo (*klf17*^*uy21*/*uy22*^) (**e**) died without hatching. Scale bar, 2 mm. After taking pictures, genotyping of individual embryos was performed by genomic PCR.

The *klf17* gene is detected in the polster during gastrulation stages and in the hatching gland during organogenesis stages^7^. We found that the *klf17*-deficient embryos (n = 19) failed to hatch at 3 days post-fertilization (dpf) and 6 dpf, whereas wild-type embryos (n = 17) hatched until 3 dpf (Figs 3 and S4). Such hatching defects were obvious and are also observed in the *klf17*-morphant^12^.

The transient *klf17*-morphant is unsuitable for continuous *in vivo* analysis. We manually removed the chorion membranes from the *klf17*-deficient embryos and grew up them. We found that the *klf17*-deficient embryos (n = 20) died without hatching approximately 15 dpf (Fig. 3). Wild-type embryos (n = 12) and the dechorionated-*klf17*-deficient embryos (n = 19) and were alive. Therefore, we examined the function of *klf17* in hatching gland development.

### Loss of hatching gland cells in the *klf17*-deficient embryos

**Figure 4:**
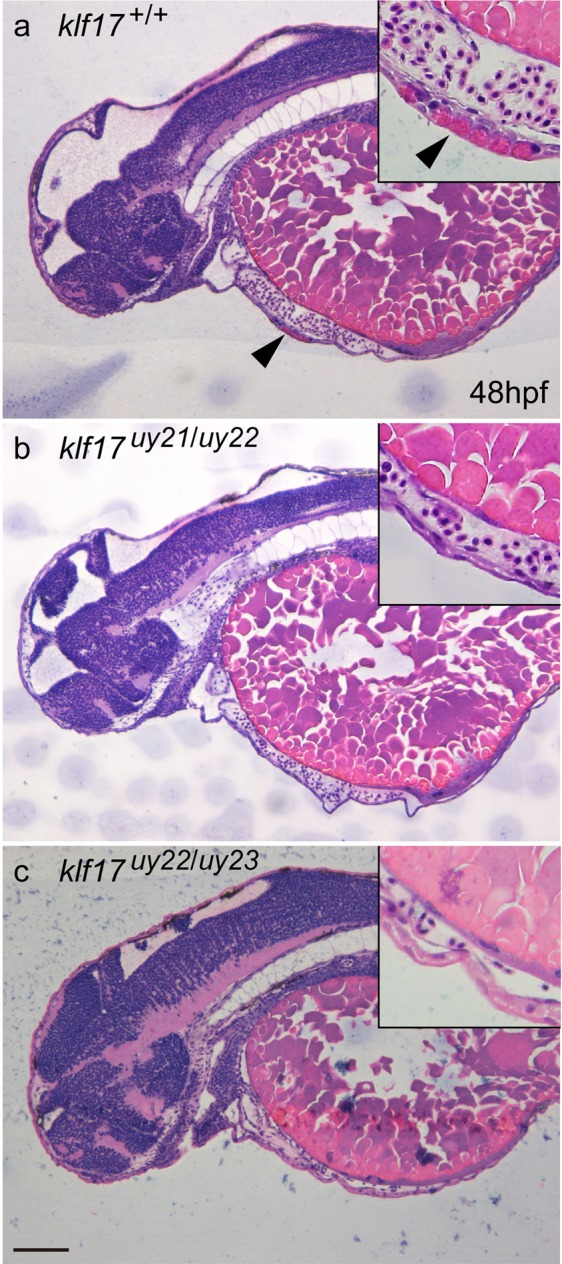
Loss of hatching gland cells in the *klf17*-deficient embryos. (**a**–**c**) Cross sections of haematoxylin and eosin (HE)-stained embryos at 48 hpf: wild-type (*klf17*^+/+^) (**a**) *klf17*-deficient embryos: *klf17*^*uy21*/*uy22*^ (**b**) and *klf17*^*uy22*/*uy23*^. (**c**) Hatching gland cells observed in wild-type embryo were missing in the *klf17*-deficient embryos. Arrowheads indicate the position of hatching gland cells. Genomic DNA was isolated from individual caudal fins, with genotyping was performed by genomic PCR. Scale bar, 100 μm.

**Figure 5:**
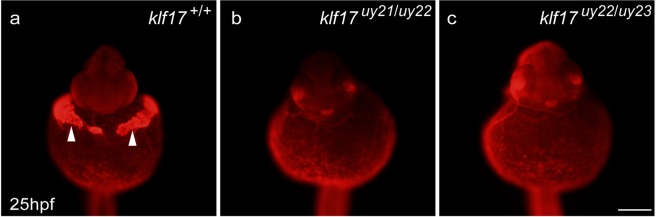
Cathepsin l 1b protein expression in the hatching gland. The expression of Cathepsin L 1b (Ctsl1b), which is one of hatching gland enzymes, was examined using whole-mount immunohistochemistry at 25 hpf. (**a**) Wild-type (*klf17*^+/+^), (**b**,**c**) *klf17*-deficient embryos: *klf17*^*uy21*/*uy22*^ (**b**) and *klf17*^*uy22*/*uy23*^. (**c**) Ventral view, anterior up. Arrowheads indicate the position of the hatching gland. After taking pictures, genotyping of individual embryos was performed by genomic PCR. Scale bar, 200 μm.

Hatching gland cells in zebrafish are located deep to the enveloping layer on the pericardial membrane. We examined the morphology of hatching gland cells using cross sections of wild-type and the *klf17*-deficient embryos at 48 hpf. Hatching gland cells visualized by haematoxylin and eosin (HE) staining were observed in wild-type embryos (n = 9), whereas the *klf17*-deficient embryos (n = 10) were completely missing the hatching gland cells (Fig. 4). Next, we examined the protein expression of Cathepsin L 1b (Ctsl1b) that is one of hatching enzymes. Using anti-Ctsl1b immunohistochemistry, Ctsl1b protein was predominantly expressed in the hatching gland cells of wild-type embryo (Fig. 5). In clear contrast to the wild-type, Ctsl1b expression was not detected in the *klf17*-deficient embryos.

**Figure 6:**
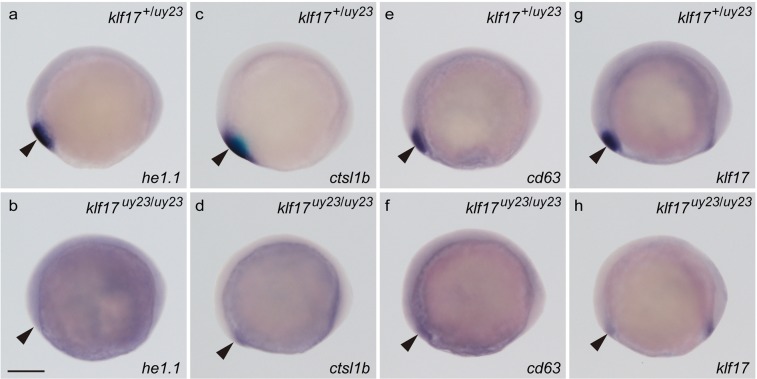
The expression of polster genes in the *klf17*-deficient embryos at the bud stage. The expression of polster markers *he1*.*1* (**a**,**b**), *ctsl1b* (**c**,**d**), *cd63* (**e**,**f**) and *klf17* (**g**,**h**) was examined by WISH at the bud stage. (**a**,**c**,**e**,**g**) Wild-type embryos (*klf17*^+/*uy23*^). (**b**,**d**,**f**,**h**) *klf17*-deficient embryos (*klf17*^*uy23*/*uy23*^). Lateral view, anterior left. Arrowheads indicate the position of the polster. After taking pictures, genotyping of individual embryos was performed by genomic PCR. Scale bar, 200 μm.

**Figure 7:**
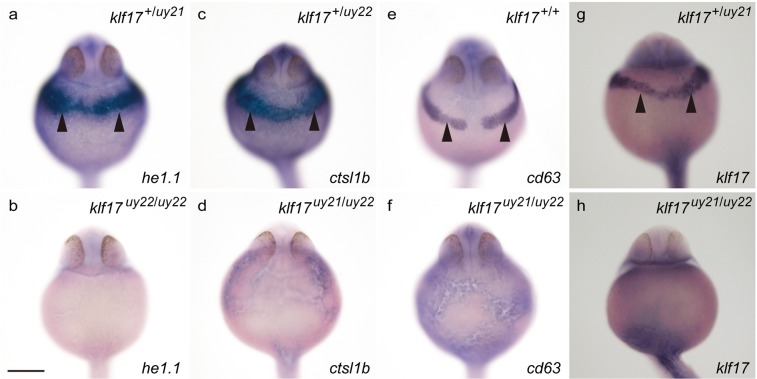
The expression of hatching gland genes in the *klf17*-deficient embryos at 25 hpf. (**a**,**c**,**e**,**g**) Wild-type embryos at 25 hpf. (**b**,**d**,**f**,**h**) *klf17*-deficient embryos at 25 hpf. Ventral view, anterior up. Arrowheads indicate the position of the hatching gland. After taking pictures, genotyping of individual embryos was performed by genomic PCR. Scale bar, 200 μm.

Next, we examined the expression of polster and hatching gland genes. Consistent with the morphological hatching gland defects, the expression of *he1*.*1* (*hatching enzyme 1*), *ctsl1b*, *cd63* and *klf17* in the *klf17*-deficient embryos was reduced in the polster of the bud stage embryos (Fig. 6), and was not detected in the hatching gland at 25 hpf (Fig. 7). We examined morphology of polster at bud stage. We found that the polster was not dected in the *klf17*-deficient embryo (Supplemental Fig. S7). These results suggested that the *klf17* gene plays important roles in the polster and hatching gland development in zebrafish.

## Discussion

Transient knockdown analysis of *klf17* suggests the involvement of *klf17* in primitive erythropoiesis and hatching gland development in zebrafish^11–13^. In this study, we have generated the *klf17*-deficient zebrafish lines and examined the physiological function of *klf17* during early embryogenesis. Although the *klf17*-morphant exhibits severe defects in primitive erythropoiesis^11,13^, both primitive erythrocytes and haemoglobin production in the *klf17*-deficient embryos were observed (Figs 1, S4 and Supplemental Movies 1–3). Reasons of these discrepancies of haematopoietic defect between the *klf17*-morphants and *klf17*-deficient embryos are not clear at present. Recent studies found that *klf1*, *klf2a*, *klf3*, *klf6a* and *klf8* in addition to *klf17* are expressed in the ICM^20^. Knockdown of *klf3* or *klf6a* induced a blockage of erythrocyte maturation. One possible explanation is that other *klf* genes expressed in the ICM function redundantly in primitive erythropoiesis in zebrafish. Multiple *klf* genes disruption based on the *klf17*-deficient zebrafish lines will be required to clarify the possibility.

Although *klf17* is known to be bilaterally expressed in lateral line neuromasts^18^, the function of *klf17* in lateral line neuromasts is not fully understood. PLL primordium migrates along the horizontal myoseptum and periodically deposits 5 or 6 neuromasts and finally forms 2 or 3 terminal neuromasts at the tip of the tail^21^. Di-ASP staining analysis revealed that the deposition of first PLL neuromast was delayed posteriorly (Fig. 2). The number of PLL neuromasts was less and the distance between first and second PLL neuromasts was short in the *klf17*-deficient embryos. The delayed deposition of PLL neuromasts was also confirmed by visualization of alkaline phosphatase accumulation in differentiated PLL neuromasts. Furthermore, the expression of *klf17* and *s100t* at 48 hpf was detected in PLL neuromasts abnormally positioned at short intervals. Because the other *klf* genes except for *klf17* are not detected in lateral line neuromasts, the induction of mutated form of *klf17* mRNA in the *klf17*-deficient embryos may affect the deposition and differentiation of PLL neuromasts. Although the molecular mechanism of PLL neuromast differentiation remains unclear, this study demonstrates that the *klf17* gene is required for proper PLL neuromast deposition.

The *klf17* is predominantly expressed in the hatching gland in zebrafish and *Xenopus*^7–9^. We found that the *klf17*-deficient embryos failed to hatch during embryogenesis (Figs 3 and S4). Such a hatching defect is consistent with that of zebrafish *klf17*-morphants and *Xenopus klf17*-morphants^13,14^. Our continuous observation revealed that the *klf17*-deficient embryos died without hatching approximately 15 dpf. Notably, the dechorionated-*klf17*-deficient embryos were alive at 15 dpf (Fig. 3). The zebrafish *klf3* gene is weakly expressed compared with the expression of *klf17* in the hatching gland, but the other *klf* genes except for *klf3* and *klf17* are not detected in the hatching gland^20^; therefore, the *klf17* would play an essential role in the hatching gland development. Using mammalian cell lines, *Klf17* was recently identified as a negative regulator of metastasis in breast cancer^6^. *Klf17* is predominantly expressed in testis and ovary in mice^4^; however, the physiological function of mammalian *Klf17* is not fully understood. Because the hatching gland does not exist in mammals, further loss-of-function analysis using *Klf17*-disrupted mice will be required to understand the developmental function of mammalian *Klf17*.

Cross section analysis of HE-stained zebrafish wild-type embryos identified the presence of hatching gland cells with cytoplasmic granules^22^, presumably containing hatching enzymes that digest the chorion membrane. In clear contrast to that analysis, the hatching gland cells were not observed in the *klf17*-deficient embryos (Fig. 4). Anti-Ctsl1b immunohistochemistry revealed that the Ctsl1b protein, which is one of hatching enzymes, was not detected in the *klf17*-deficient embryos (Fig. 5). Furthermore, the expression of polster and hatching gland marker genes, *he1*.*1*, *ctsl1b* and *cd63* was reduced in the *klf17*-deficient embryos at the bud stage and 25 hpf (Figs 6 and 7). The polster at the bud stage was not detected in the *klf17*-deficient embryo. Therefore, the *klf17* gene is an indispensable transcription factor for the polster and hatching gland development in zebrafish. If the physiological function of the *klf17* gene is conserved among fish, the disruption of fish *klf17* genes mediated by genome editing technologies may be useful for eliminating invasive alien fish in closed areas.

## Methods

### Ethics statement

All animal experiments were performed in accordance with the animal protocol approved by the Institutional Animal Care and Use Committee (IACUC) of the University of Yamanashi. The IACUC of the University of Yamanashi approved this study (Approval Identification Number: A25–28).

### Synthetic crRNA and tracrRNA, recombinant Cas9 protein and microinjection

To disrupt the targeted *klf17* genomic locus, we used the ready-to-use CRISPR/Cas9 system composed of CRISPR RNA (crRNA), *trans*-*activating* crRNA (tracrRNA) and recombinant Cas9 protein^23^. Synthetic crRNAs and tracrRNA (Supplementary Table S1), and recombinant Cas9 protein were obtained from the Integrated Device Technology, Inc (IDT). Synthetic *klf17*-crRNA1 (25 pg), *klf17*-crRNA2 (25 pg) and tracrRNA (100 pg) were co-injected together with recombinant Cas9 protein (1 ng) into 1-cell stage zebrafish embryos. Klf17-morpholinos (*klf17*-MO, 5′-TGCAAATGTTAGGGAACTCAGAAGG-3′) were injected at one-cell stage embryos as described previously^24^. *klf17* mRNA (20 pg) was injected into blastomere at one-cell stage embryos.

### Genotyping for the *klf17* locus and genomic sequencing

To prepare the genomic DNA, the embryos at indicated stages were incubated in 108 μl of 50 mM NaOH at 98 °C for 10 min. Subsequently, 12 μl of 1 M Tris-HCl (pH 8.0) was added to the solution^23^. Genomic fragments at the targeted sites were amplified by PCR with PrimeTaq (GENETBIO Inc.) and the locus-specific primers are listed in Supplementary Table S2. The PCR conditions were as follow: 40 cycles of 98 °C for 10 s, 55 °C for 30 s and 72 °C for 30 s. To perform heteroduplex mobility assay (HMA) for genotyping, the resultant PCR amplicons were electrophoresed on a 12.5% polyacrylamide gel^25^. To confirm individual mutations, genomic fragments for the targeted genomic locus were amplified from the solution (1 μl) using PCR (Supplementary Table S2). The resultant PCR fragments were sub-cloned into the pGEM-T Easy vector (Promega) and genomic sequences were determined by sequence analysis.

### Lateral line neuromast labeling

Lateral line neuromasts at the 54 hpf stage embryos were labeled by incubation of live fish with 0.5 mM 4-(4-diethylaminostyryl)-N-methylpyridinium iodide (4-Di-2-ASP) in E3 (5 mM NaCl, 0.17 mM KCl, 0.33 mM CaCl_2_ and 0.33 mM MgSO_4_) medium for 7 min. Labelled fish were washed 3 times with E3 medium and anaesthetized with tricaine (3-amino benzoic acidethylester), and subsequently were observed under fluorescence microscope. For lateral line neuromast labeling with alkaline phosphatase^26^, the embryos at 54 hpf were fixed in 4% paraformaldehyde for 3 h at room temperature and washed with phosphate buffered saline plus 0.1% Tween-20 (PBST). The embryos were developed in alkaline phosphate buffer containing NBT and BCIP (Nacalai tesque) for 30 min.

### Histological analysis

Embryos were dehydrated in a graded series of ethanol and embedded using a Technovit 8100 kit (Kulzer). Embedded embryos were sectioned on a Leica RM2125 microtome at 6 μm and mounted on slides. Embryos were stained with haematoxylin-eosin (HE) after sectioning.

### Whole-mount immunohistochemistry

Embryos were incubated with anti-Cathepsin L 1b (GeneTex, Inc.) at 4 °C overnight in PBST containing 5% sheep serum and washed 4 times with PBST. Subsequently, embryos were incubated with Alexa Fluor 594 goat anti-rabbit IgG (Invitrogen) at room temperature for 4 h in PBST containing 5% sheep serum and washed 4 times with PBST.

### Whole-mount *in situ* hybridization (WISH)

We examined the expression of *s100t*^27^, *he1*.*1*, *ctsl1b*^28^, *cd63*^29^, *klf17*, *gata1*, *β*_e3_*globin* and *lyz*. Whole-mount *in situ* hybridization (WISH) was performed as previously described^30^. Zebrafish embryos hybridized with the digoxigenin (DIG)-labelled RNA probe were incubated with alkaline phosphatase-conjugated anti-DIG antibody. To visualize the RNA probe recognized by the anti-DIG antibody, the embryos were subsequently incubated with BM Purple (Roche) as the substrate. Washing the embryos with PBST terminated the colour reaction, and the embryos were fixed in 4% paraformaldehyde.

## Supplementary information

Supplemental Movie 1

Supplemental Movie 2

Supplemental Movie 3

Supplemental Information
