# Supplementary material for: Characterization of biklf/klf17-deficient zebrafish in posterior lateral line neuromast and hatching gland development
Source: Sci Rep. 2019 Sep 26;9:13680. doi: 10.1038/s41598-019-50149-5 (PMC6763433; doi:10.1038/s41598-019-50149-5)
Supplement: Supplementary file 4 — Supplemental Information [file 41598_2019_50149_MOESM4_ESM.pdf]

Characterization of *biklf/klf17*-deficient zebrafish in posterior lateral line neuromast and hatching gland development

Hiroaki Suzuki[1*], Tomoe Ishizaka[1*], Kanoko Yanagi[1], Ryota Sone[1], Yuto Sunaga[2], Rie Ohga[1], Atsuo Kawahara[1]

[1]Laboratory for Developmental Biology, Center for Medical Education and Sciences, Graduate School of Medical Science, University of Yamanashi, Shimokato 1110, Chuo, Yamanashi, 409-3898, Japan

[2]Department of Pediatrics, Faculty of Medicine, University of Yamanashi, Shimokato 1110, Chuo, Yamanashi, 409-3898, Japan

[*]These authors contributed equally to this work.

Correspondence and requests for materials should be addressed to A.K. (e-mail: akawahara@yamanashi.ac.jp).

Supplementary Information

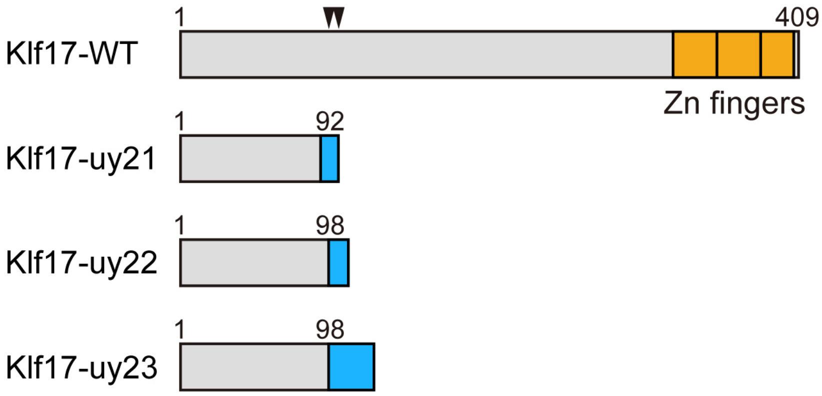

Figure S1. Representation of the predicted molecular structure of wild-type and mutant Klf17 proteins. The $klf17^{uy21}$, $klf17^{uy22}$ and $klf17^{uy23}$ lesions cause premature stop codons after 11, 13 and 29 missense amino acids (blue rectangles) starting at amino acid 92, 98 and 98, respectively. Zinc-finger domains are indicated in orange rectangles. The arrowheads indicate the CRISPR/Cas9 target sites.

biklf wild-type

TGACAACGACAACACCTCAGCACCGCCGCCCGCCTATTCCCTCCCGGAATCACCGGAGAGCTGC
AGCACCGTGTATGACAGCGATGGATGTCACCCGACCCCTAACGCCTACTGTGGCA

biklf uy21 50bp deletion and 30bp insertion: total 20bp deletion

TGACAACGACAACACCTCAGCACCGCTGCACCGCTCAGCATTCCTGCAGCACCGTGTATGACGA
TGGATGTCACCCGACCCCTAACGCCTACTGTGGCA

biklf uy22 41bp deletion and 21bp insertion: total 20bp deletion

TGACAACGACAACACCTCAGCACCGCCGCCCGCCTATTCCCTCAGCACCGCCGCCCGCCTATTT
TGGATGTCACCCGACCCCTAACGCCTACTGTGGCA

biklf uy23 20bp deletion and 48bp insertion: total 28bp insertion

TGACAACGACAACACCTCAGCACCGCCGCCCGCCTATTCCCTTCATCTCCCGGGTGAATCACCG
GAGAGCTGCAGCACCGTGTATGACAGCTCCAGCTCTCTGGTCACTGGTCGGGTGACATCCATCC
CCTAACGCCTACTGTGGCA

Figure S2. Nucleotides sequences of wild-type and mutant *klf17* genes. Underlines indicate the targeted sequences for biklf-HMA-F1 and biklf-HMA-R1 primers. Blue letters indicate the targeted sequences for *klf17*-crRNA1 and *klf17*-crRNA2, while green letters indicate PAM (protospacer adjacent motif) sequences. Red letters indicate inserted sequences.

Klf17-WT

MALADAMLPSINTFSNNHILDEKQSEIVRDWKVDIAKTNPRAGDVRPLIE
VEFSIVESPPLAKDEDDLSKFLDLEFILSNTVTSDNDNTSAPPPAYSLPE
SPESCSTVYDSDGCHPTPNAYCGTNFNSRPGHSLVAELFTPDMNYQGEYN
LKGHLDRLEYTELRALNTRNQQHLTNSNNAGYKIKTENQEQSCMMVNDYM
GHYYAQEPQRVMQHQTYGHHVQQDVPRDILGRKDCILTEMNTQHHIDISH
QQQFINNAHFPPQYAQHQQYHGHFNMFSEPLRANHPAMPGVMLTPPSSPL
LGFLSPEDSKPKRGRRSWARKRTATHSCEFPGCGKTYTKSSHLKAHMRTH
TGEKPYHCSWEGCGWKFARSDELTRHYRKHTGHRPFQCHLCERAFSRSDH
LALHMKRHM

Klf17-uy21

MALADAMLPSINTFSNNHILDEKQSEIVRDWKVDIAKTNPRAGDVRPLIE
VEFSIVESPPLAKDEDDLSKFLDLEFILSNTVTSDNDNTSAPLHRSAFLQ
HRV

Klf17-uy22

MALADAMLPSINTFSNNHILDEKQSEIVRDWKVDIAKTNPRAGDVRPLIE
VEFSIVESPPLAKDEDDLSKFLDLEFILSNTVTSDNDNTSAPPPAYSLST
AARLFWMSPDP

Klf17-uy23

MALADAMLPSINTFSNNHILDEKQSEIVRDWKVDIAKTNPRAGDVRPLIE
VEFSIVESPPLAKDEDDLSKFLDLEFILSNTVTSDNDNTSAPPPAYSLHL
PGESPESCSTVYDSSSSSLVTGRVTSIP

Figure S3. Amino acids sequences of wild-type and mutant Klf17 proteins. Red letters indicate missense amino acids.

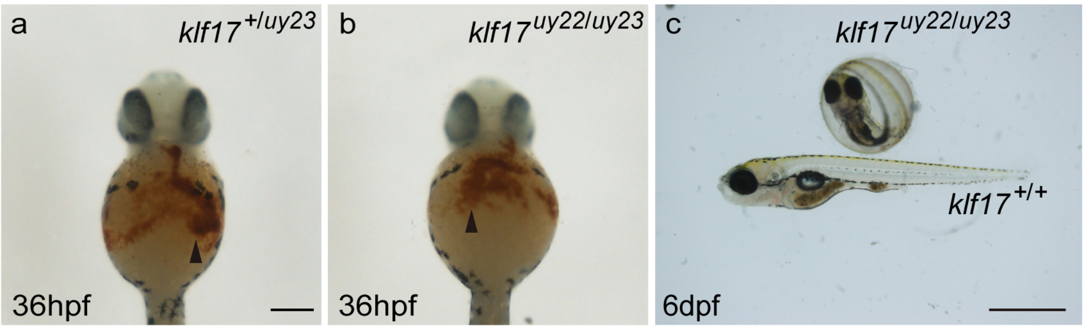

Figure S4. *klf17*$^{uy22/uy23}$ embryos failed to hatch during zebrafish embryogenesis. (a, b) Haemoglobin production (arrowheads) in wild-type (*klf17*$^{+/uy23}$) and the *klf17*-deficient embryo (*klf17*$^{uy22/uy23}$) at 36 hpf (ventral view, anterior up). Scale bar, 200 µm. (c) Hatching-deficient phenotype in the *klf17*-deficient embryo (*klf17*$^{uy22/uy23}$) at 6 dpf. Scale bar, 1 mm. After taking pictures, genotyping of individual embryos was performed by genomic PCR.

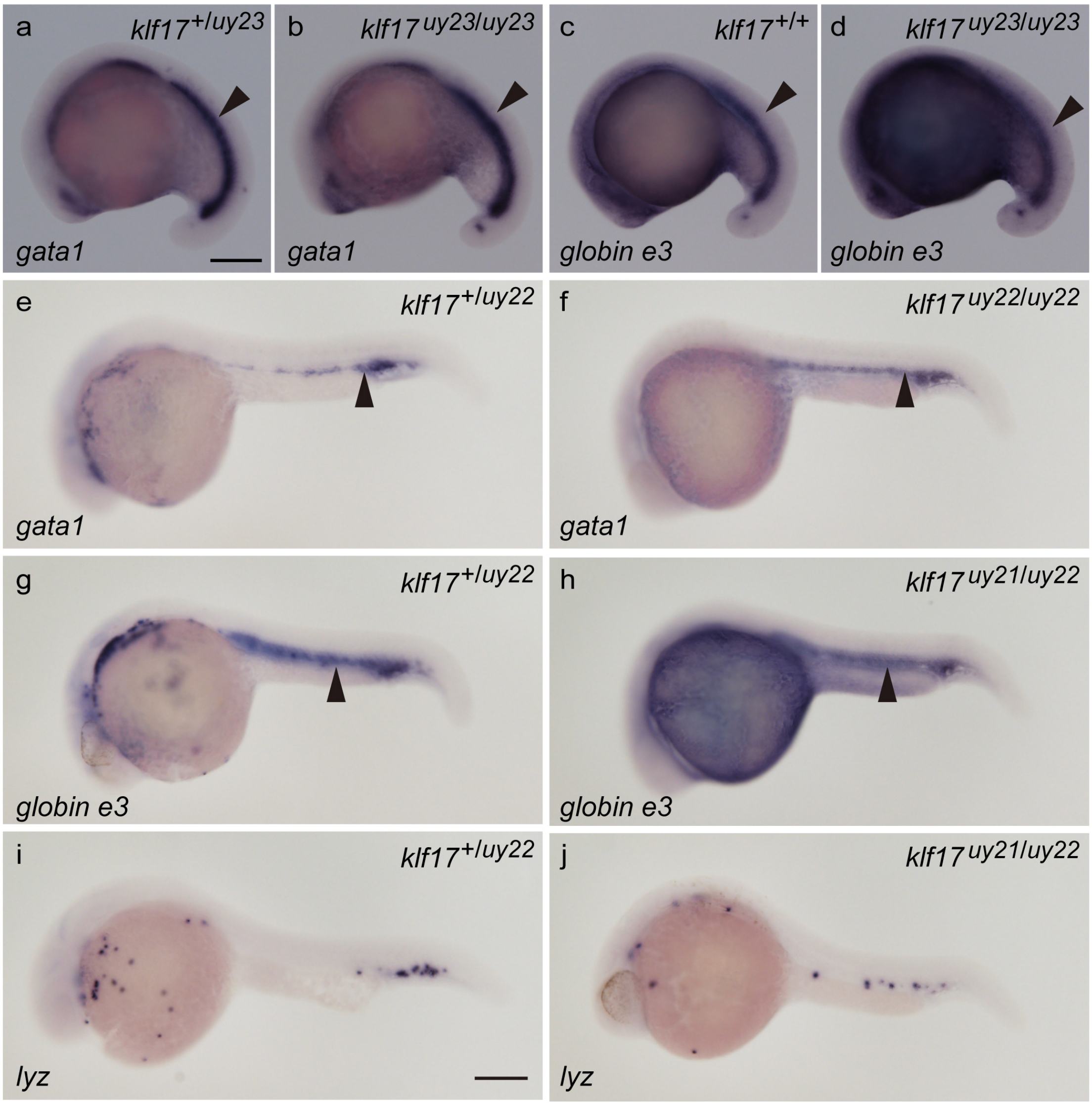

Figure S5. The expression of haematopoietic genes in the *klf17*-deficient embryos. The expression of haematopoietic markers, *gata1* (a, b, e, f), β_{e3}*globin* (c, d, g, h) and *lysozyme C* (*lyz*) (i, j), was examined by WISH at 18-somite stage (a-d) or at 25 hpf (e-j). (a, c, e, g, i); wild-type embryos. (b, d, f, h, j); *klf17*-deficient embryos. Lateral view, anterior left. Arrowheads indicate the position of the intermediate cell mass. After taking pictures, genotyping of individual embryos was performed by genomic PCR. Scale bar, 200 μm.

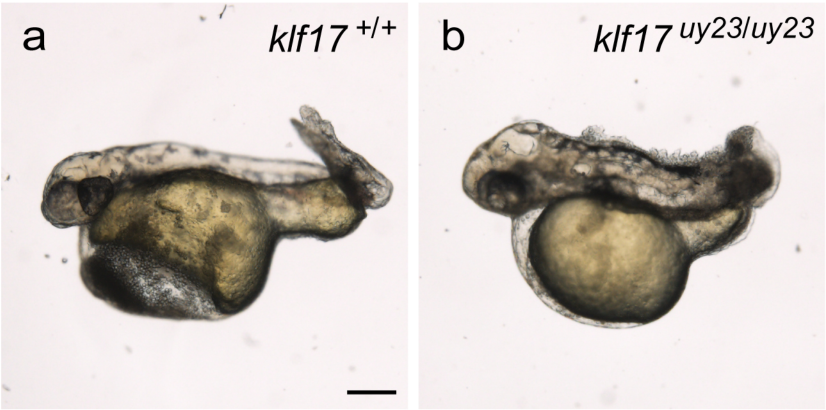

Figure S6. Injection of *klf17* mRNA into wild-type and *klf17*-deficient embryos. (a) *klf17* mRNA-injected wild-type embryo at 36 hpf. (b) *klf17* mRNA-injected *klf17*-deficient (*klf17*$^{uy23/uy23}$) embryo at 36 hpf. *klf17* mRNA (20pg) was injected into blastomere at one-cell stage embryos. Scale bar, 200 μm. After taking pictures, genotyping of individual embryos was performed by genomic PCR.

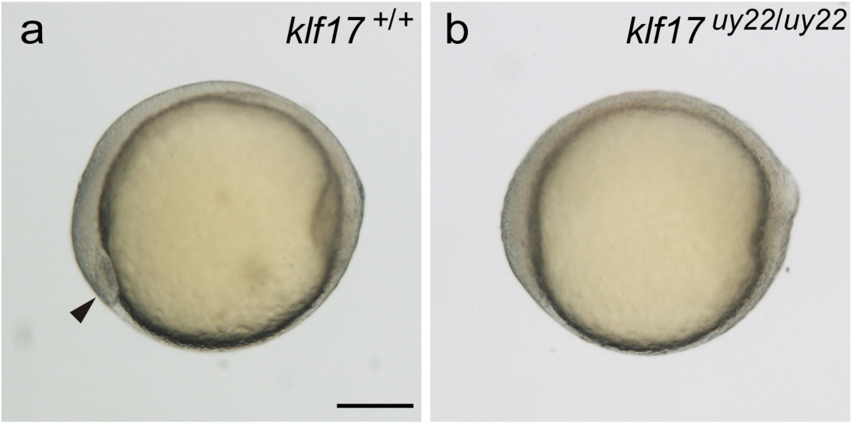

Figure S7. Morphology of polster in wild-type and *klf17*-deficient embryos. (a, b) Morphology of polster in wild-type (*klf17*$^{+/+}$) and *klf17*-deficient embryo (*klf17*$^{uy22/uy22}$) embryos at bud stage (lateral view, anterior left). The polster at the bud stage was not detected in the *klf17*-deficient embryo. Arrowhead indicates position of the polster. Scale bar, 200 μm. After taking pictures, genotyping of individual embryos was performed by genomic PCR.

Supplemental Movie 1. Blood circulation of wild-type embryo at 36 hpf.

Supplemental Movie 2. Blood circulation of *klf17*$^{uy22/uy22}$ embryo at 36 hpf.

Supplemental Movie 3. Blood circulation of *klf17*$^{uy22/uy23}$embryo at 36 hpf.

Table S1. The targeted genomic sequences.

Targeted genomic sequences for CRISPR/Cas9

Target	Sequence (5’ to 3’)
klf17-1	GCTCTCCGGTGATTCCGGGAGGG
klf17-2	AGCACCGTGTATGACAGCGATGG

PAM sequences are underlined.

Table S2. PCR primers used in this study.

Primer name	Sequence (5’ to 3’)
biklf-HMA-F1	TGACAACGACAACACCTCA
biklf-HMA-R1	TGCCACAGTAGGCGTTAGG
